# Unprovoked internal jugular vein thrombosis: a case report and literature review

**DOI:** 10.1186/s12959-020-00246-7

**Published:** 2021-01-06

**Authors:** Loïc Payrard, Léa Iten, Jacques Donzé, Gregor John

**Affiliations:** 1 Department of Internal Medicine, Neuchâtel Hospital Network, Rue de la Maladière 45, CH-2000 Neuchâtel, Switzerland; 2 Department of Internal Medicine, University of Lausanne, Lausanne, Switzerland; 3 Brigham and Women’s Hospital, Harvard Medical School, Boston, MA USA; 4 Department of Internal Medicine, Geneva University Hospitals (HUG), Gabrielle-Perret-Gentil 4, CH-1205 Geneva, Switzerland

**Keywords:** Internal jugular vein, Thrombosis, Thrombophilia, Unprovoked

## Abstract

**Background:**

Managing thrombosis in rare sites is challenging. Existing studies and guidelines provide detailed explanations on how to overcome lower-limb thromboses and pulmonary embolisms, but few studies have examined thrombosis in rare sites. Lack of data makes clinical practice heterogeneous. Recommendations for diagnosing, treating, and following-up internal jugular vein thrombosis are not clearly defined and mostly based on adapted guidelines for lower-limb thrombosis.

**Case presentation:**

A 52-year-old Caucasian woman came to the Emergency Department with chest, neck, and left arm pain. Computed tomography imagery showed a left internal jugular vein thrombosis. An extensive workup revealed a heterozygous factor V Leiden gene. Therapy was initiated with intravenous unfractionated heparin, then switched to oral acenocoumarol, which resolved the symptoms. Based on this case presentation and a literature review, we summarize the causes, treatment options, and prognosis of unprovoked internal jugular vein thrombosis.

**Conclusions:**

Managing internal jugular vein thrombosis lacks scientific data from large randomized clinical trials, partly because such thromboses are rare. Our literature review suggested that clinical treatments for internal jugular vein thrombosis often followed recommendations for treating lower-limb thrombosis. Future specific studies are required to guide clinicians on the modalities of diagnosis, screening for thrombophilia or oncologic disease, treatment duration, and follow-up.

## Background

Thromboembolic disease is the third most frequent cardiovascular disease [1]. It has been thoroughly studied in recent decades, resulting in a standard diagnostic strategy, international guidelines, and new medications [2, 3].

There is scarce medical literature available on unprovoked internal jugular vein (IJV) thrombosis, essentially just case reports and short case series. IJV thrombosis is, therefore, often managed with reference to guidelines dedicated to thromboses occurring in more common sites, notably the deep veins of the lower limbs [2, 3]. Yet unusual thrombosis sites are associated with distinct risk factors and complications, and some treatments (e.g. direct oral anticoagulants) have not been tested specifically. Thus, we believe that specific recommendations are needed to guide the treatment management of patients with thromboses in uncommon sites.

This report describes a case of unprovoked IJV thrombosis and an accompanying literature review about diagnosing and treating this condition.

## Case presentation

A 52-year-old Caucasian woman under treatment for hypothyroidism arrived at the Emergency Department with constrictive chest pain that had been radiating into her left arm and cervical region for 1 week, accompanied by new-onset dyspnea which had worsened 2 days before her medical visit. Her physical examination was unremarkable, except for high blood pressure (188/104 mmHg) and excess weight (body mass index = 28 kg/m2); a blood test showed a high D-dimer concentration (2170 μg/l). A computed tomography (CT) scan performed to exclude pulmonary embolism was inconclusive. However, fat infiltration around the left jugular-carotid led to a further investigation using neck ultrasound. This showed a thrombosis emerging from the base of the left subclavian vein and extending 11 centimeters into the IJV, sparing the cerebral vessels.

The patient described spontaneous hair loss and a 10 kg gain in weight over the previous year. She was taking levothyroxine but took no other medication or hormonal substitutes. There was no history of smoking, recent surgery, trauma, infectious disease, intravenous medical or recreational drug use, or past catheter insertion.

A chest CT scan and a colonoscopy revealed no cancer. A recent gynecological check-up consisting of a physical examination, a mammography, and a Pap smear found no pathology. A thyroid-stimulating hormone test and urinary cortisol were normal. Because of the patient’s young age and no obvious risk factor for thromboembolic disease, we completed the investigations with a thrombophilia workup. Antinuclear antibodies were positive at 1:320, but without other criteria suggesting overt lupus [4]. Otherwise, antinucleoprotein antibodies (SSA, SSB, RNP, Sm, Scl70, Jo1), antinucleosome antibodies, anticardiolipin antibodies, and anti-B2-glycoprotein antibodies were negative. We did not test for lupus anticoagulant due to the patient’s anticoagulation treatment and the risk of a false-positive [5]. Antithrombin, protein C, and protein S activity were within the normal ranges and were tested before the introduction of anticoagulant treatment. Only a heterozygous factor V Leiden gene (R506Q) was found positive. We concluded on the diagnosis of an unprovoked IJV thrombosis.

After 5 days of intravenous unfractionated heparin, symptoms rapidly regressed, allowing a switch to an oral anticoagulant (acenocoumarol). The patient was discharged home 1 week after admission. After 3 months, the decision to continue anticoagulation with acenocoumarol was taken according to European Society of Cardiology guidelines [2] for patients over 50 years old with unprovoked thrombosis and a low risk of major bleeding (HAS-BLED score for major bleeding risk = 1 due to hypertension, corresponding to a 3.4% risk [6]). 6 months after Emergency Department admission, the patient was symptom-free.

## Discussion and conclusions

We have described a patient presenting with unprovoked IJV thrombosis, heterozygous factor V Leiden gene, and no other obvious risk factors. This rare thrombosis site reveals the limits of international guidelines based on large, randomized clinical trials mainly designed for deep-vein thrombosis in a limb or pulmonary embolism. There is, therefore, a lack of evidence which might guide clinicians in their workup and treatment of thrombosis in rare sites. Although, by their very nature, published case reports and case series provide biased information, exploring similar cases can be a first step (and sometimes the only one possible) towards collecting scientific evidence.

### Medline research strategy

We searched Medline using the keywords (*spontaneous OR unprovoked OR idiopathic OR primary AND internal jugular vein thrombosis AND English*) and excluded reports of provoked IJV thrombosis to collect information on causes, treatment modalities, and complications. We retrieved 40 relevant articles in English (Table 1). These articles included 48 patients with unprovoked IJV from 17 countries, but mostly from Japan (9/40 articles). More than a third (17/48; 35%) were still truly idiopathic after the workup (Table 2).

**Table 1:** Case reports and case series of unprovoked internal jugular vein thrombosis

Author, journal, year [ref]	Country	Nb^**a**^	Age	Gender (F/M)	Thrombosis location	Associated factors	Imag-ing	Symptoms	Treatment (type and length)	Complication
Payrard et al. 2020	Switzerland	1	52	F	Left IJV		US + CT	Painful neck swelling	Intravenous heparin for 5 days and acenocoumarol prolonged	/
Algoblan et al., *Cureus*, 2020 [7]	Saudi Arabia	1	66	M	Right IJV, subclavian and axillary vein	Liver transplant	US + CT	Right chest, flank and back pain	Enoxaparin 1 mg/kg twice daily	/
Pratt et al., *J Thromb Thrombolysis*, 2020 [8]	USA	1	21	F	Left IJV, transverse and sigmoid sinus	Nitrous oxide abuse	MRI	Confusion, hallucinations, weakness and falls	LMWH (duration not describe)	/
Agrawal et al., *Cureus*, 2019 [9]	USA	1	64	F	Right IJV and subclavian vein	/	US	Right upper extremity and right facial swelling	Intravenous heparin for 2 days and Apixaban for 6 months	/
Hahn et al., *Auris Nasus Larynx*, 2019 [10]	Germany	3/41^a^	50, 54, 78	2 F, 1 M	Internal jugular vein (side not describe)	3 idiopathic, 22 paraneoplastic (compression and distant disease), 14 inflammatory diseases, 2 central-venous catheter	US + CT	Asymptomatic	Apixaban 5 mg (duration not describe) / Rivaroxaban 20 mg for 2 months / Rivaroxaban for 3 months	/
Matsuda et al., *eNeurologicalSc*i, 2018 [11]	Japan	1	71	M	Right IJV	Pseudotumor cerebri and Lung cancer	Veinography + US	Not describe	Warfarin, 3 mg/day	/
Al-Zoubi, *Vasc Health Risk Manag*, 2018 [12]	Jordan	1	44	F	Right IJV	Antiphospholipid syndrome	US + CT	Painful neck swelling	LMWH for 3 months and long-term warfarin	/
Jendoubi, *Saudi J Anaesth*, 2017 [13]	Saudi Arabia	1	75	M	Right IJV	/	US	Exacerbation of COPD	Not describe	Pulmonary embolism
Toratani et al., *Intern Med*, 2017 [14]	Japan	1	45	M	Left IJV subclavian and brachiocephalic vein	Gastric cancer	US + CT	Swelling of the left side of his neck and left upper limb	Unfractionated heparin for 7 days and long-term Edoxaban (60 mg/day, orally)	/
Nomura et al., *Intern Med*, 2016 [15]	Japan	1	70	F	Left IJV	/	CT	Dyspnea	Anticoagulation therapy for 2 weeks	Pulmonary embolism
Bandara et al., *J Med Case Rep*, 2016 [16]	Sri Lanka	1	75	M	Left IJV, external jugular and brachiocephalic vein	Prostate carcinoma	US + CT	Painless swelling of the left supraclavicular fossa	Intravenous heparin and long-term oral warfarin	/
Efe et al., *Perfusion*, 2015 [17]	Turkey	1	19	F	Right IJV	/	US + CT + MRI	Pain and swelling of the right side of the neck	Enoxaparin and warfarin for 6 months	/
Onishi et al., *Case Rep Nephrol Dial*, 2015 [18]	Japan	1	56	M	Left IJV and subclavian vein	Nephrotic syndrome	CT	Swelling of the upper limb	Heparin and warfarin (duration not describe)	/
Van den Broek et al., *Neth J Med*, 2014 [19]	Netherlands	1	28	F	Right IJV	Borderline ovarian tumour	US + MRI	Mass in the right neck	Tinzaparin 0.9 ml daily (duration not describe)	/
Altintas et al., *Case Rep Vasc Med*, 2014 [20]	Turkey	1	42	F	Bilateral IJV	Inherited coagulopathy (homozygous MTHFR mutation and protein C deficiency)	US + MRI	Asymptomatic	LMWH and long-term warfarin	Recurrent thrombosis (lower limb DVT)
Ghatak et al., *J Anaesthesiol Clin Pharmacol*, 2013 [21]	India	1	21	F	Right IJV and subclavian vein	Homozygous Factor V Leiden mutation, activated factor C resistance and Dengue infection	US	Septic shock and encephalopathy	Unfractionated heparin and warfarin (duration not describe)	/
Papay et al., *J Crohns Colitis*, 2013 [22]	Austria	1/157^a^	NA	NA	IJV (side not describe)	Inflammatory bowel disease	Not describe	Not describe	Not describe	Recurrent thrombosis (venous arm thrombosis)
Kunimasa et al., *Intern Med*, 2013 [23]	Japan	1	70	F	Left IJV	Lung cancer (Trousseau’s syndrome)	CT	Painful swelling left side of the neck	Not describe	/
Honma et al., *J Echocardiogr*, 2011 [24]	Japan	1	59	F	Left internal jugular vein	/	US + CT	Dizziness	Oral warfarin (duration not describe)	/
Ishida et al., *J Neurosurg Spine*, 2011 [25]	Japan	1	56	F	Left internal jugular vein	Cervical spontaneous spinal epidural hematoma	MRI + Angiography	Pain of the back of the head and neck	/ (because of the hematoma)	/
Gbaguidi et al., *QJM*, 2011 [26]	France	5/29^a^	NA	NA	18/29 Left IJV 7/29 Right IJV 4/29 Bilateral IJV	24/29: secondary IJV 5/29: idiopathic IJV	US + CT	Cervical oedema, arm oedema, pain, erythrocyanosis, superficial varicose collateral veins	2 UFH, 7 LMWH, followed by oral anticoagulation (20), Median duration: 6 months	3/29: pulmonary embolism (secondary IJV) 12/29: post thrombotic syndrome (secondary IJV)
Serinken et al., *Kaohsiung J Med Sci*, 2010 [27]	Turkey	1	31	F	Right IJV	/	US + CT	Painless swelling in the right anterior side of the neck	LMWH and oral warfarin	/
Snijders et al., *Eur J Gynaecol Oncol*, 2010 [28]	Netherlands	1	52	F	Right IJV	Non-hodgkin’s lymphoma of both ovaries	US + CT	Swelling of the ride side of the neck	UFH and oral coumarin (duration not describe)	/
Handley et al., *Int J Otolaryngol*, 2010 [29]	UK	1	30	M	Right IJV vein and sigmoid sinus	Collet-Sicard Syndrome secondary to IJV thrombosis	CT + MRI	Right sided neck pain, dysphagia and dysphonia	Not describe	/
Leibman et al., *J Emerg Med*, 2009 [30]	Israel	1	31	F	Right IJV	Ovarian hyperstimulation syndrome	US	Pain and swelling of the right side of the neck	LMWH (duration not describe)	/
Chlumsky et al., *Acta Cardiol*, 2009 [31]	Czech Republic	1	61	M	Right IJV	/	US	Swelling of the right side of the face	LMWH and warfarin for 6 months	/
Pata et al., *J Laryngol Otol*, 2008 [32]	Turkey	2	58, 46	F, M	Left IJV	Breast and lung cancer	MRI / CT	Painful swelling of the left side of the neck / Painless mass of the left neck and dyspnea	UFH + oral VKA (duration not described) / No treatment because patient died of cancer	/
Mori et al., *Clin Appl Thromb Hemost*, 2008 [33]	Japan	1	16	F	Left IJV, brachiocephalic and subclavian vein	Primary Mediastinal Large B-Cell lymphoma (Trousseau’s syndrome)	CT	General fatigue and dyspnea	Dalteparin (duration not describe)	/
Kikuchi et al., *Br J Radiol*, 2004 [34]	Japan	1	56	M	Left IJV and external jugular vein	/	CT + FDG-PET	Swelling around the left parotid gland	/ (treated with antibiotics)	/
Cheang et al., *J Laryngol Otol*, 2004 [35]	UK	1	42	M	Bilateral IJV	Malignant lymphadenopathy	US + CT	Diffuse swelling and stiffness of the neck	UFH (type and duration not describe)	/
Unsal et al., *Eur Arch Otorhinolaryngol*, 2003 [36]	Turkey	1	48	M	Right IJV	Lung and prostate cancer	US + CT	Pain and swelling of the right side of the neck	UFH and oral coumarin (duration not describe)	/
Khandekar et al., *Angiology*, 2003 [37]	India	1	30	M	Bilateral IJV	Protein S Deficiency	CT	Swelling of face, neck and both upper limbs	Anticoagulation + aspirin 150 mg/day	/
Thomas et al., *Blood Coagul Fibrinolysis*, 2001 [38]	UK	1	29	F	Right IJV	Protein S deficiency	US	Pain of the right side of the neck	UFH and Dalterapin 6000 UI twice daily + aspirin 75 mg and Dalteparin 5000 UI twice daily (duration not describe)	/
Van den Noortgate, *Acta Clin Belg*, 2000 [39]	Belgium	1	88	F	Right IJV	Colorectal adenocarcinoma	US + CT	Pain and swelling of the side of the neck	LMWH and oral anticoagulation (type not describe) for 3 months	/
Todros et al., *Hum Reprod*, 1999 [40]	Italy	1	30	F	Left IJV, subclavian, axillary and humeral veins	Ovarian hyperstimulation syndrome	US	Pain and swelling of the left arm and neck	Heparin (2 weeks) and warfarin (13 weeks) and back to heparin (5 weeks)	/
Kalan et al., *J Laryngol Otol*, 1996 [41]	UK	1	65	M	Left IJV	Squamous cell carcinoma (primary lesion not found)	US + CT	Hoarseness	Heparin (type not describe) for 2 months (stopped because patient died)	Death (related or not to IJV thrombosis?)
Holland et al., *Aust N Z J Surg*, 1996 [42]	Australia	1	21	F	Right IJV	Antiphospholipid syndrome	CT	Pain and swelling of the right side of the neck	Heparin (type not describe) and long-term oral warfarin	/
Hines et al., *Gynecol Oncol*, 1995 [43]	USA	1	49	F	Left IJV + subclavian vein	Ovarian and endometrial adenocarcinoma	US + CT + MRI	Pain and swelling of the left side of the neck	Heparin + warfarin (duration not describe)	Pulmonary embolism
Langlieb et al., *Gynecol Oncol*, 1992 [44]	USA	1	46	F	Left IJV	Leiomyosarcoma of the omentum	CT	Pain of the left side of the neck	Not describe	Not describe
Carrington et al., *Postgrad Med J*, 1988 [45]	UK	2	56, 59	F, M	1 Right IJV 1 Left IJV	Invasive carcinoid and mesothelioma	CT	Painful and neck swelling	Heparin and acenocoumarol (duration not describe)	Patient with left IJV: extension to the left subclavian vein
Kennedy et al., *Ann Otol Rhinol Laryngol*, 1987 [46]	USA	1	77	F	Left IJV	/	CT	Nagging sensation of fullness in the inferior left side of her neck	Heparin (type not describe) for 1 week and oral warfarin for 3 months	/

Search results = 197 (on 31.03.2020). Advanced Search Builder: spontaneous OR unprovoked OR idiopathic OR primary AND internal jugular vein thrombosis AND English[lang] Sort by: Best Match. Exclusion criteria: article not directly related to the topic. Exclusion’s criteria: papers non-directly linked with the topic, after death discovery, pediatric cases (< 16 years old), non-English papers

*NA* not assessed, *LMWH* Low molecular weight heparin, *UFH* unfractionated heparin, *VKA* vitamin K anticoagulation

^a^ Number of cases of idiopathic cases

**Table 2:** Etiologies of patients (*N* = 48) diagnosed with unprovoked IJV (based on a literature review)

Etiology	Number (%)
Idiopathic	17 (35.4)
Paraneoplastic disease	16 (33.3)
Coagulopathy	4 (8.3)
Ovarian hyperstimulation syndrome (OHS)	2 (4.2)
Antiphospholipid syndrome	2 (4.2)
Other causes	7 (14.6)

### Internal jugular vein thrombosis epidemiology

Upper-limb thrombosis accounts for a maximum of 10% of all deep-vein thromboses [2, 47]. IJV thrombosis is even less frequent, but epidemiological studies are lacking (other unusual sites for thrombosis, such as the mesenteric vein, account for 0.002–0.006% of all inpatient admissions) [48]. Overall, the most common causes of IJV thrombosis are cancer, central venous catheter placement, and ovarian hyperstimulation syndrome (OHS) [26]. IJV thrombosis can be provoked or unprovoked. The former accounts for four out of five cases [26]. Most IJV thromboses are secondary to central catheter or pacemaker placement, extrinsic compression (by a tumor or malformation), local infections (e.g., Lemierre disease), cervical trauma, or ENT cancer. Unprovoked IJV thrombosis is uncommon and has been associated with paraneoplastic disease, thrombophilia, OHS, and idiopathic IJV [10, 26, 49–51]. OHS is associated with thromboembolic complications, but their mechanisms are incompletely understood [52]. In addition to the usual hemostatic changes during pregnancy [53], OHS can cause increased levels of coagulation factors [54].

### Clinical presentation

The spectrum of potential clinical presentations is broad, ranging from asymptomatic disease to diffuse and non-specific pain. Neck pain and swelling are the most frequent symptoms, found in 24/48 patients (50%). IJV thrombosis is a serious event, with a potentially fatal outcome, but complications in IJV thrombosis have seldom been reported [55]. Pulmonary embolism was reported in 3 cases (6%). Surprisingly, there were no cases describing neurological complications. The risk of recurrent IJV thrombosis is unknown since the follow-up of published cases was generally interrupted after 3 to 6 months (Table 1).

### Diagnostic testing

D-dimer concentrations have excellent negative predictive value, even for deep-vein thrombosis of the upper extremities. However, D-dimer concentrations have not been validated with IJV thrombosis because of the lack of prospective outcome trials. Lower D-dimer sensitivity in IJV thrombosis could be an issue, resulting in higher false-negative rates, which could lead to fatal consequences [56, 57].

Performance of imaging to diagnosis IJV is unknown. The majority of reports used ultrasonography often completed with CT (21/48; 43.8%). In cases of unprovoked IJV thrombosis, oncological disease and thrombophilia should be screened for since they are more common than in cases of lower-body thrombosis. Indeed, these conditions are frequently associated with IJV thrombosis (Table 2).

Thrombophilia is defined as a hereditary or acquired genetic abnormality predisposing the patient to thromboembolic events [58]. The F5 R506Q gene (the factor V Leiden mutation) is the most common pro-thrombotic gene mutation in Caucasians [59], affecting 3–7% of this population. It is a gain-of-function mutation in the procoagulant factors. The annual incidence of venous thromboembolism (VTE) in heterozygous carriers of the factor V Leiden mutation is approximately 0.45% [60].

Middeldorp et al. recommended not routinely screening for hereditary thrombophilia as it does not affect most patients’ clinical management, and long-term anticoagulation is generally recommended for patients with unprovoked thrombosis [58]. Thrombophilia screening is only recommended for young (age < 50) patients with a VTE, in cases of recurrent VTE, and in cases involving a family history of VTE [61]. Hereditary thrombophilia does not significantly increase the risk of recurrence (relative risk from 1.4–2.5, depending on the type of mutation [61]), and anticoagulation treatment should be stopped after 3 months in the absence of risk factors such as cancer. American guidelines also propose anticoagulation cessation after 3 months [3]. European guidelines propose individual assessments of the risks of recurrence and major bleeding in order to decide on whether anticoagulation treatment should continue [2]. In the present case, we chose to screen for thrombophilia and cancer because the thrombosis was unprovoked and located in an unusual site. The patient’s borderline age was also taken into account in accordance with an algorithm proposed by Connors [5].

In our review, only 8% (4/48) of patients with IJV thrombosis had an inherited coagulopathy, a low prevalence also seen among Caucasians if patients from Japan, Sri Lanka, India, Jordan, Israel, Turkey, and Saudi Arabia are excluded from the review. Paraneoplastic disease was far more common than inherited coagulopathy, affecting one third of patients (16/48). Thus, an active workup for oncological diseases is of the utmost importance in unprovoked IJV thrombosis.

### Treatment of IJV thrombosis

There are no specific guidelines for the treatment of IJV thrombosis. Treatment is often based, by analogy, on guidelines [2, 3] for upper-limb thrombosis (Tables 1 and 3). Initial treatment (5–21 days following diagnosis) consists of parenteral therapy with low molecular weight heparin (or unfractionated heparin) with a transition using vitamin K antagonists. The alternative therapy for non-cancer patients is high-dose direct oral anticoagulants. Treatment duration is at least 3 months, with possible long-term anticoagulation depending on the cause of thrombosis [2]. To date, no randomized clinical trials have evaluated anticoagulation for upper extremity deep-vein thrombosis. The only ongoing research is a prospective clinical study assessing oral apixaban for the treatment of upper extremity deep-vein thrombosis (including IJV thrombosis). However, to the best of our knowledge, no results have been published yet [62]. The majority of cases in our literature review used unfractionated heparin (26/40; 65%), usually in association with an oral anticoagulant (19/40; 47.5%). Treatment duration varied from 2 weeks to 6 months for non-paraneoplastic cases (Table 3).

**Table 3:** Patient characteristics from the literature review (see text for details)

Median age	51 years old (16–88 y.o.)
Sex	F: 26/48 (54.2%)
	M: 16/48 (33.3%)
	Unknown: 6/48 (12.5%)
Number of countries	17
Modality of diagnostic imaging	US: 7/48 (14.6%)
	CT: 11/48 (22.9%)
	US + CT: 19/48 (39.5%)
	US + MRI: 2/48 (4.2%)
	US + CT + MRI: 2/48 (4.2%)
	Other or unknown modality: 7/48 (14.6%)
Type of treatment	Heparin only: 7/40 (17.5%)
	Heparin + oral anticoagulant: 19/40 (47.5%)
	Oral anticoagulant only: 3/40 (7.5%)
	Other or unknown: 11/40 (27.5%)
Type of oral anticoagulant	Vitamin K antagonists: 18/22 (81.8%)
	Direct oral anticoagulants: 3/22 (13.6%)
	Unknown: 1/22 (4.6%)
Treatment duration	3 months: 3/48 (6.25%)
	6 months: 3/48 (6.25%)
	Long-term: 5/48 (10.4%)
	Other: 4/48 (8.3%)
	Unknown: 33/48 (68.8%)
Complications	Pulmonary embolism: 3/48 (6.25%)
	Neurological complication: 0/48 (0%)
	Recurrent thrombosis: 2/48 (4.15%)
	Extension of thrombosis: 1/48 (2.1%)
	No complication: 42/48 (87.5%)

*Abbreviations*: *F* female, *M* male, *US* ultrasound, *CT* computed tomography, *MRI* magnetic resonance imaging

### Follow-up of IJV thrombosis

No specific guidelines exist for IJV thrombosis follow-up. Gbaguidi et al. used a venous ultrasound scan at three and 6 months. Boedeker et al. [55] proposed an ultrasound scan each month until 6 months of follow-up.

The management of internal jugular vein thrombosis is heterogenous and currently based on the management of lower extremity deep-vein thrombosis, with a low rate of complications. The lack of guidelines and large series means that the modalities of diagnosis and treatment type and duration are variable. Given the low prevalence of IJV thrombosis, large, randomized studies would be hard to carry out. Data from our literature review suggest that treatments could tend towards those used for lower limb deep-vein thrombosis, but that the modalities of diagnosis and the duration of treatment and follow-up should be clarified.

## Data Availability

All data generated or analyzed during this study are included in this published article.

## Abbreviations

ARDS: Acute respiratory distress syndrome
BID: Bowel inflammatory disease
COPD: Chronic obstructive pulmonary disease
CT: Computed tomography
ENT: Ear, nose, and throat
IJV: Internal jugular vein
Jo1: Antihistidyl transfer-RNA synthetase antibodies
MRI: Magnetic resonance imaging
NSCLC: Non-small cell lung cancer
OHS: Ovarian hyperstimulation syndrome
RNP: Antiribonucleoprotein antibodies
SSA: Anti-Sjögren’s syndrome type A antibodies
SSB: Anti-Sjögren’s syndrome type B antibodies
Scl70: Anti-scleroderma antibodies
Sm: Anti-Smith antibodies
US: Ultrasound
VTE: Venous thromboembolism
